# Restoration of Defective CFTR in Human Nasal Respiratory Epithelial Cells by CFTR Modulators and mRNA Transfection

**DOI:** 10.3390/ijms27042063

**Published:** 2026-02-23

**Authors:** Roshani Narayan Singh, Marilia Marta Horn, Marin Juko, Ami Kampshoff, Jochen Schmid, Heymut Omran, Dandan Zhang, Joseph Rosenecker, Wolf-Michael Weber, Jörg Große-Onnebrink

**Affiliations:** 1 Department of General Paediatrics, University Hospital Muenster, Albert-Schweitzer-Campus 1, 48149 Muenster, Germany; 2 Institute of Molecular Microbiology and Biotechnology, University of Muenster, Corrensstr. 3, 48149 Muenster, Germany; 3 CCRC Hauner—Comprehensive Childhood Research Centre, Ludwig-Maximilians University Munich, Lindwurmstr. 4, 80337 Munich, Germany

**Keywords:** cystic fibrosis, modulators, mRNA treatment, Ussing chamber, immunofluorescence, mucus viscosity

## Abstract

The cystic fibrosis transmembrane conductance regulator (CFTR) is a member of the atypical ATP-binding cassette (ABC) family that functions as a phosphorylation-regulated epithelial anion channel. Cystic fibrosis (CF) is characterised by variants in the CFTR gene that lead to impaired epithelial chloride–ion transport and increased mucus viscosity. Although CFTR modulators such as Trikafta^®^ have transformed the care of many CF patients, individuals harbouring rare CFTR variants still have no effective treatment options. In this study, we used primary air–liquid interface (ALI) airway cultures obtained from 21 CF patients (pwCF) and 21 healthy controls (HC) to evaluate the therapeutic efficacy of CFTR restoration based on chitosan-mediated CFTR mRNA and modulators. While modulators restored CFTR channel function in most cultures derived from CF patients, those with class I or other rare variants showed no improvement. Chitosan-mediated CFTR mRNA delivery successfully restored CFTR function in ALI cultures of patients carrying rare CFTR variants with limited or no observed clinical response to modulator therapy, assessed by electrophysiology using our newly developed Multi Transepithelial Current Clamp (MTECC) Ussing chamber. This was then confirmed by morphological visualisation of CFTR protein expression in modulator-responsive patient samples using immunofluorescence (IF) staining. IF revealed an increase in CFTR signal and the restoration of epithelial barrier integrity following chitosan-mRNA and modulator treatment as a secondary outcome alongside CFTR functional measurements. Notably, MUC5AC expression, a major gel-forming mucin expressed by airway goblet cells and mucus viscosity were elevated in CF cultures, but were markedly reduced following successful intervention, approaching the levels seen in HCs. These findings establish the potential of chitosan-mRNA delivery as a therapeutic approach for CF patients, particularly those who do not respond to modulators. They also provide a practical, comparative evaluation of advanced mRNA-based treatments in patient-derived airway models.

## 1. Introduction

Cystic fibrosis (CF) is a life-limiting autosomal recessive disease affecting over 70,000–100,000 individuals worldwide, with most cases resulting in chronic respiratory insufficiency and reduced life expectancy. However, prevalence varies substantially by ancestry and region and the actual number of patients may well be much higher [1]. Caused by mutations in the cystic fibrosis transmembrane conductance regulator (CFTR) gene, CF is characterised by disrupted chloride and bicarbonate secretion at epithelial surfaces, leading to thickened, viscous airway mucus, impaired mucociliary clearance, and increased susceptibility to recurrent pulmonary infections [2]. Although survival for patients with CF (pwCF) has steadily improved over recent decades, due to advances in symptomatic therapies, airway clearance regimens, and infection management, disease progression remains a challenge, particularly for pwCF lacking access to variant-specific treatments like CFTR modulators.

The CFTR (ABCC7) is a unique member of the ABC transporter superfamily because it functions as an ATP-gated ion channel rather than an active transporter. While most ABC proteins use energy from the hydrolysis of ATP to actively transport substrates such as lipids, peptides or drugs across membranes, CFTR uses ATP binding and hydrolysis to control the opening and closing of its chloride and bicarbonate permeation pathway [3]. This unusual mechanism distinguishes CFTR as the only real ion channel within the ABC family, highlighting its dual nature as both a channel and a central regulatory protein in epithelial ion transport. Dysfunction of the CFTR is the underlying cause of CF and contributes to several other pathophysiological conditions, emphasising its pivotal role in epithelial homeostasis and its broader significance in the study of membrane transporters in health and disease [4].

A crucial therapeutic breakthrough has been the development of CFTR small-molecule modulators, such as the triple combination regimen Trikafta^®^ (elexacaftor/tezacaftor/ivacaftor; ETI) [5]. By targeting the underlying molecular defects in the most prevalent CFTR mutations, these agents can significantly enhance CFTR channel function at the airway surface, translating into improvements in lung function, nutritional status, and quality of life for a large proportion of patients. CFTR modulators fall into two main categories based on their mechanism of action: correctors, which improve the folding and trafficking of CFTR proteins to the apical membrane, and potentiators, which increase the open probability of the channel once it is present at the surface. Contemporary combination therapies (e.g., ETI) exploit this dual mechanism by increasing the amount of mature CFTR protein at the membrane and simultaneously enhancing its gating function [6].

However, a substantial subset that is up to 10–15% of all patients carry CFTR variants that are non-responsive to approved modulator therapies [7,8]. For these individuals, highly personalised approaches, readily scalable to both common and modulator non-responsive variants, are urgently needed. Furthermore, despite substantial improvement in CFTR function with CFTR modulator therapy, CFTR activity does not reach physiological levels, which allows residual airway inflammation to persist and thereby may contribute to ongoing lung disease. Therefore, the evaluation of novel therapies aimed at improving CFTR function is essential.

mRNA-based therapeutics provide a highly promising strategy for genetic diseases like CF, as they can restore functional protein by delivering a wild-type transcript regardless of the patient’s specific mutation [9]. Efficient, safe delivery of mRNA to airway epithelial cells remains a significant hurdle. Lipid nanoparticles (LNPs), widely used in mRNA vaccine development, have shown potential for nucleic acid transport across biological barriers, but their translation to the pulmonary epithelium remains hampered by challenges in tissue targeting, inability to overcome the mucus barrier, uptake, immunogenicity, and long-term safety. Chitosan, a naturally occurring polysaccharide derived from chitin, demonstrates excellent biocompatibility, mucoadhesive properties, and a naturally available non-immunogenic profile for mucosal applications in the respiratory tract [10,11]. Chitosan-based mRNA nanocomplexes (NCs) offer distinct mechanistic advantages for pulmonary delivery—including enhanced mucus retention, reversible tight junction modulation, efficient endosomal escape, and reduced lipid-driven immunogenicity—making them particularly well suited for airway gene restoration strategies in CF [10]. Moreover, chitosan-mRNA NCs can be delivered easily via nebulisation of aerosols [12].

Reliable evaluation of new mRNA therapeutics in CF airways mandates functional and morphological analyses using primary patient-derived cells, which authentically reflect the diverse genetic background and pathological hallmarks of human CF. Comparative studies that examine modulator-responsive and non-responsive patient samples can establish therapeutic value for both common and rare variants, a crucial benchmark for emerging therapies. Complementary endpoints, such as Ussing chamber measurements of CFTR-mediated transepithelial ion transport, IF staining of the CFTR and tight junction proteins (e.g., Claudins) might help to overcome the existing problems [13]. Early clinical trials of inhaled mRNA therapy for cystic fibrosis have demonstrated an acceptable safety profile, but clinical efficacy has been limited and variable, largely due to inefficient delivery of the therapy across the thick mucus that lines CF airways, and the transient nature of mRNA expression, which requires repeated dosing. These challenges highlight the need for optimised pulmonary delivery systems that are specifically adapted to the unique biophysical and inflammatory environment of the CF airway. One example of this is chitosan.

To conduct studies involving modulators or mRNA transfection, a Ussing chamber system that enables sterile working conditions is required, allowing the cells to be measured multiple times. The MTECC (Multi Transepithelial Current Clamp) system, which we have developed and established, has proven itself in this regard [14]. Moreover, the MTECC system combines the precision of classical Ussing chamber analysis with higher sensitivity, reproducibility, and throughput, while being better suited for personalised CFTR function testing in human airway epithelia. Additionally, rheological assessment of mucus viscosity and composition are essential for robust mechanistic validation.

Biological fluids, like blood and mucus, exhibit non-Newtonian behaviour under normal physiological conditions. It means they show a non-linear viscosity dependency on shear stress or shear rate changes. In the respiratory system, bronchial mucus, which is moved by actions like coughing or beating of cilia, shows shear-dependent viscosity that differs in both healthy- and pathological-patient systems. The composition of mucus varies, but primarily consists of up to 95% water, along with mucin (2–5%), lipids (1–2%), and salts (about 1%) [15]. The mucins, which are large glycoproteins, play the leading role in providing structural integrity of the three-dimensional gel-like arrangement established by an entangled network. This intrinsic internal structure mainly contributes to the non-Newtonian or shear-thinning behaviour of bronchial mucus [16]. Additionally, it influences flow of mucus through airways and allows its function in trapping and clearing foreign particles or pathogens that are eventually degraded by enzymes and eliminated.

Genetic diseases can directly impact mucus composition, and consequently, its viscosity behaviour. Conditions such as CF alter the mucus aspect, primarily due to inadequate hydration that leads to a significant increase in viscosity. This impairs the normal clearance of mucus from the lungs, which potentially facilitates colonisation and infection of the lungs by bacteria [17]. Therefore, a thorough understanding of mucus rheology is essential for advancing treatments for CF and other chronic respiratory conditions.

This study aims to compare the efficacy of CFTR restoration using approved CFTR modulators and chitosan-mediated wild-type CFTR (wtCFTR) mRNA delivery in patient-derived ALI airway cultures. Using electrophysiological, molecular and rheological analyses, we seek to determine whether mRNA-based therapy could restore CFTR function and epithelial homeostasis, particularly in patients carrying non-responsive modulator variants. Our findings highlight the translational promise of chitosan-based wtCFTR-mRNA delivery for patients not approved for current modulator therapy, providing an adaptable pathway toward personalised CFTR restoration in CF.

## 2. Results

### 2.1. cAMP Activation of CFTR in Human Nasal Epithelia

ALI filters were placed in the MTECC containing Ringer’s solution on either side of confluent pseudostratified epithelia derived from HC and pwCF (for a detailed list of patient’s variants see Supplementary Materials, S1). We recorded continuously and simultaneously transepithelial potential (PD) and transepithelial resistance (R_t_). From these values, we calculated the transepithelial conductance (G_t_). The addition of a cAMP cocktail containing a membrane-permeable cAMP analogue (100 µM) and IBMX (1 mM) to the basolateral side activated CFTR, as measured by an increase in G_t_. IBMX is a potent phosphodiesterase inhibitor and prevents the degradation of cAMP. Some of the conductance values are negative, yet this is the result of the mathematical difference between the G_t_ values in the presence and absence of cAMP, since sometimes R_t_ slightly increases.

**Figure 1:**
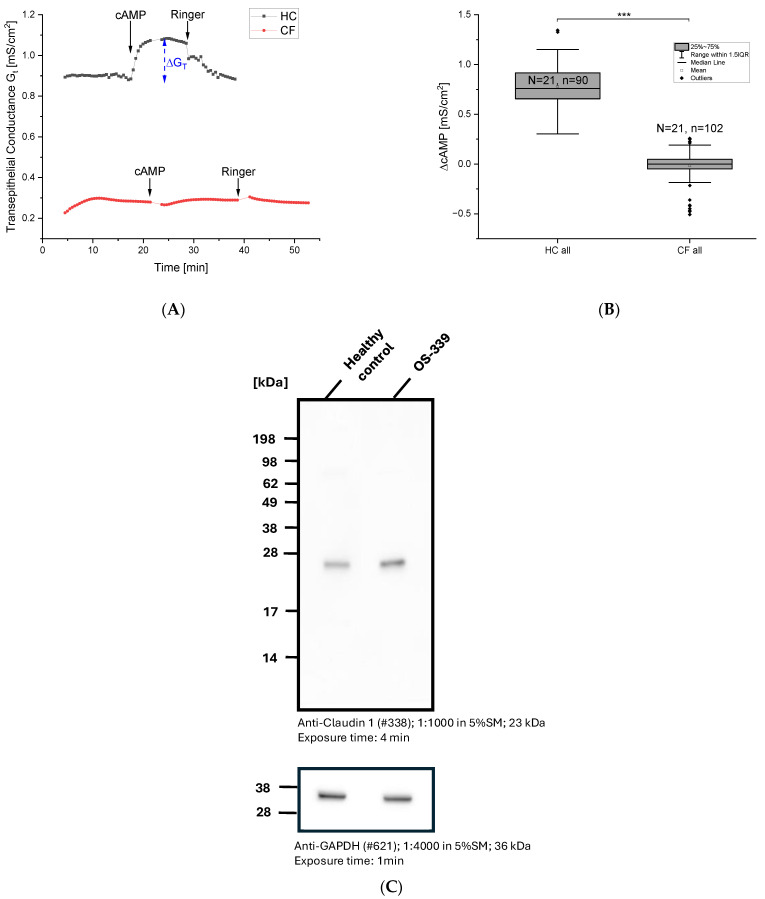
(**A**) Representative time course of transepithelial measurements of ALI cultures from HC and from pwCF cells. Shown is the averaged G_t_ (in mS/cm^2^) of four filters in a typical measurement using the MTECC system. HC cells (black trace) showed an increase in G_t_ after application of the cAMP cocktail (100 µM cAMP, 1 mM IBMX). Cells from pwCF (red trace) display a generally lower G_t_, which did not respond to cAMP cocktail. The blue arrow in the upper trace indicates how ΔG_t_ is determined for all cases described herein. (**B**) Statistical analyses of G_t_ in HC and cells from pwCF. Boxes indicate the interquartile range (IQR), with the horizontal line showing the median. Whiskers extend to 1.5 IQR; data points beyond this range are shown as diamonds and represent statistically defined outliers. G_t_ in HC cells is significantly higher (*p* ≤ 0.001 (***)) than in pwCF cells. N = number of donors, n = number of filters. CF donors (N = 21; 13 females, 8 males). HC samples (N = 21; 15 females, 6 males) were primarily obtained from volunteer students and practitioners who provided verbal consent for nasal cell sampling. In accordance with ethical standards and institutional guidelines, no personal or clinical data were recorded or evaluated, and all samples were used in anonymised form. (**C**) Western blot analysis showed stronger expression of claudin-1 in cells from a pwCF (F508del homozygous) compared with HC. GAPDH was used as loading control to confirm equal protein loading and comparable GAPDH expression between HC and pwCF samples. For densitometric quantification of the Western blot please refer to Supplementary Materials, S2.

Figure 1A shows a typical time course of a measurement with cells from HC (upper black trace) and cells derived from pwCF (lower red trace). At the times indicated by the arrows we basolaterally applied the cAMP cocktail. HC cells responded with a marked increase in G_t_ on the cAMP cocktail, indicating activation of the CFTR. Subsequent wash-out of cAMP brought G_t_ back to its initial value. G_t_ values in presence and absence of cAMP were used to calculate ΔcAMP values. However, application of the cAMP cocktail to cells from pwCF showed no detectable response of G_t_, demonstrating defective CFTR function. Statistical analyses of the cAMP responses in cells of HC and pwCF showed significantly lower cAMP-induced G_t_ (ΔcAMP) values for cells from pwCF (Figure 1B); while cells from healthy subjects exhibited a G_t_ of 0.825 ± 0.025 mS/cm^2^, cells from pwCF had significantly lower G_t_ of −0.015 ± 0.015 mS/cm^2^. These data demonstrate that MTECC measurements allow a clear distinction between ALI cultures derived from nasal brushings of healthy subjects compared to cells derived from pwCF. As previously reported, a threshold value of approximately 0.3 mS/cm^2^ was found for ΔcAMP; HC cells always exceed this value, whereas cells from pwCF never reach it in our investigations.

Since G_t_ is a reciprocal value of R_t_, these data confirm and extend our previous observations that CF respiratory epithelia exhibit elevated resistance as compared to HC [14]. Figure 1C shows a typical Western blot with higher expression of claudin-1 in cells from pwCF. In CF ALI cultures without inflammatory injury, these primary epithelial changes result in an increased tight junctional barrier with higher R_t_, indicating altered paracellular ion selectivity rather than enhanced barrier quality [18]. Western blot results were confirmed by densitometric analysis using ImageJ (Supplementary Materials, S2).

### 2.2. Influence of Modulators on CFTR Function

CFTR modulators have transformed the therapeutic landscape of CF by targeting the underlying protein defect directly. Correctors such as tezacaftor and elexacaftor improve the folding, trafficking and stability of misfolded CFTR variants, particularly the common F508del mutation. Potentiators such as ivacaftor enhance the probability that the CFTR channel will open at the cell surface. Combination regimens are increasingly achieving substantial restoration of CFTR function in vitro and in vivo, as evidenced by improved CFTR activation [14], epithelial hydration and clinical outcome measures. Due to the significant impact of these modulators on epithelial ion transport and cellular morphology, it is crucial to develop reliable in vitro methods that can accurately measure their efficacy on CFTR function in patient-derived airway cultures.

**Figure 2:**
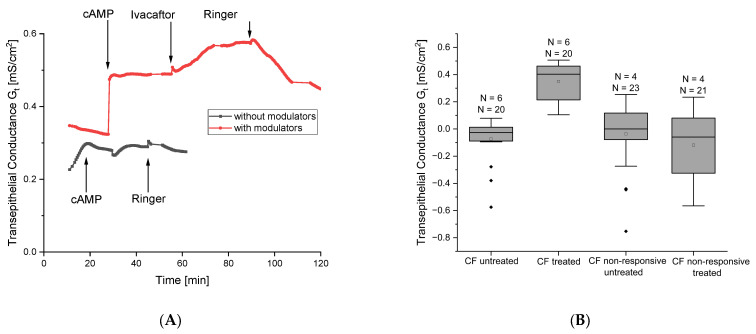
(**A**) Representative time course of transepithelial measurements of cells from patients with cystic fibrosis in absence and presence of modulators. Cells from pwCF (F508del/R553X) showed no response to the cAMP cocktail in absence of modulators (black trace) while the same cells showed marked response to cAMP and ivacaftor after preincubation with modulators tezacaftor and elexacaftor (red trace). (**B**) Statistical analyses of cells from pwCF carrying different genetic variants. Boxes indicate the interquartile range (IQR), with the horizontal line showing the median. Whiskers extend to 1.5 IQR; data points beyond this range are shown as diamonds and represent statistically defined outliers.

We examined the effects of incubation with tezacaftor and elexacaftor on ALI cell cultures from pwCF after first determining the cAMP response of these cells (Figure 2A). As expected, the cells from pwCF exhibited low mean G_t_ values (0.227 mS/cm^2^) and showed minimal or no response to cAMP activation (Figure 2A, black trace). After 72 h of incubation with correctors, we measured the cells again and, in addition to the cAMP response, tested the cells’ response to ivacaftor. The initial mean G_t_ value of the incubated cells from pwCF (0.347 mS/cm^2^) was significantly higher than the previously measured values. In addition, the cAMP response after treatment with correctors was clearly pronounced and led to activation of the CFTR, which could be further enhanced by the application of ivacaftor (Figure 2A, red trace). The functional readout showed that approximately 30 percent responded with at least partial functional restoration of the CFTR.

In Figure 2B, we summarise and statistically evaluate the modulator results. Six of the pwCF ALI cultures examined responded to incubation with tezacaftor and elexacaftor with a significant improvement in G_t_ values. Donors in this cohort had at least one F508del allele. However, the cells of four pwCF showed no response to incubation with correctors, meaning they belong to the class of genetic variants that are not suitable for modulator therapy. The left part (responsive patients) lists patients who are suitable for modulator therapy, while the right part of the figure lists patients who carry genetic variants that make the use of modulators inadvisable (non-responsive patients).

### 2.3. Altered MUC5AC Expression in CF Airway Epithelial Cultures Is Reduced in Modulator-Responsive Patients

**Figure 3:**
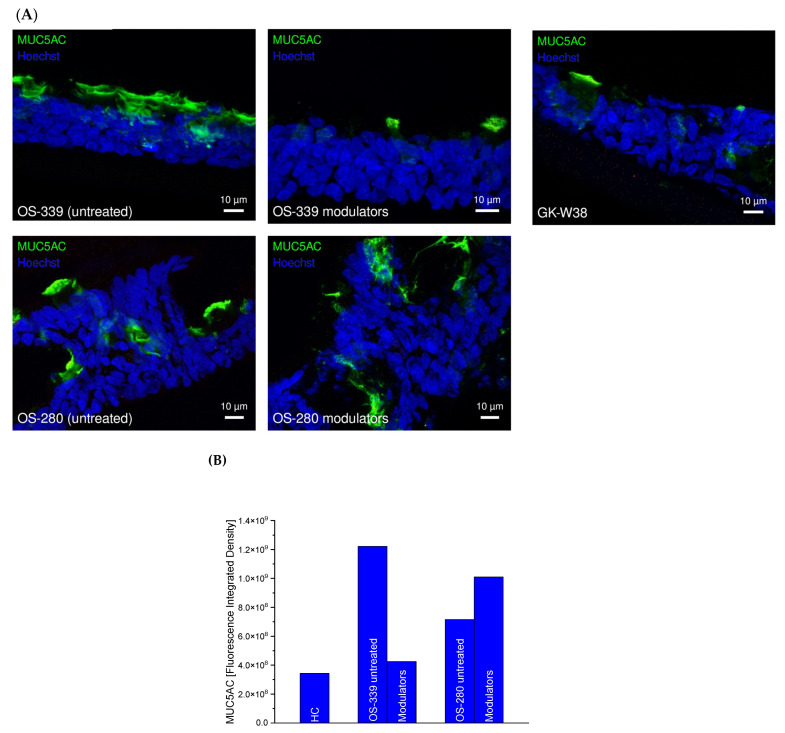
MUC5AC expression in ALI cultures from pwCF with different CFTR genotypes. (**A**) Representative immunofluorescence images showing MUC5AC (green, 1:500) and nuclei (Hoechst 33342, blue, 1:1000) in HC (GK-W38) and pwCF ALI cultures. OS-339 is CFTR ΔF508 homozygous (modulator-responsive), whereas OS-280 carries the rare CFTR variant c.1898+3A>G homozygous (modulator-non-responsive). Cultures were analysed under untreated conditions and 72 h post-CFTR modulator treatment. (**B**) Quantification of MUC5AC fluorescence (integrated density, a.u.). Modulator treatment reduces MUC5AC levels in the responsive ΔF508 homozygous sample (OS-339) but not in the non-responsive rare-variant sample (OS-280). Scale bars are 10 µm.

MUC5AC is a highly specific marker of the differentiated respiratory epithelium, reflecting the identity of goblet cells and their capacity for mucin production [19]. Its restricted expression within the airway mucosa, together with its predominant apical localisation, enables reliable identification of secretory epithelial subpopulations and provides a functional correlate of mucociliary activity and epithelial remodelling. We therefore assessed MUC5AC expression in airway epithelial ALI cultures derived from HC and pwCF before and after incubation with CFTR modulators (Figure 3).

Under basal conditions, CF ALI cultures exhibited increased MUC5AC expression compared with HC, consistent with a hypersecretory epithelial phenotype [20]. Importantly, incubation with CFTR correctors elexacaftor and tezacaftor resulted in a reduction in MUC5AC expression in CF cultures derived from modulator-responsive patients (OS-339; ΔF508 homozygous). In contrast, no marked changes in MUC5AC expression were observed in cultures derived from pwCF carrying modulator-non-responsive variants, (OS-280: c.1898+3A>G homozygous). These findings suggest that restoration of CFTR function by modulator treatment is associated with secondary normalisation of mucus-associated epithelial alterations only in responsive CF airway epithelia (Figure 3).

### 2.4. Restoration of Defective CFTR in CF Human Nasal Epithelial Cells by wtCFTR-mRNA Transfection

**Figure 4:**
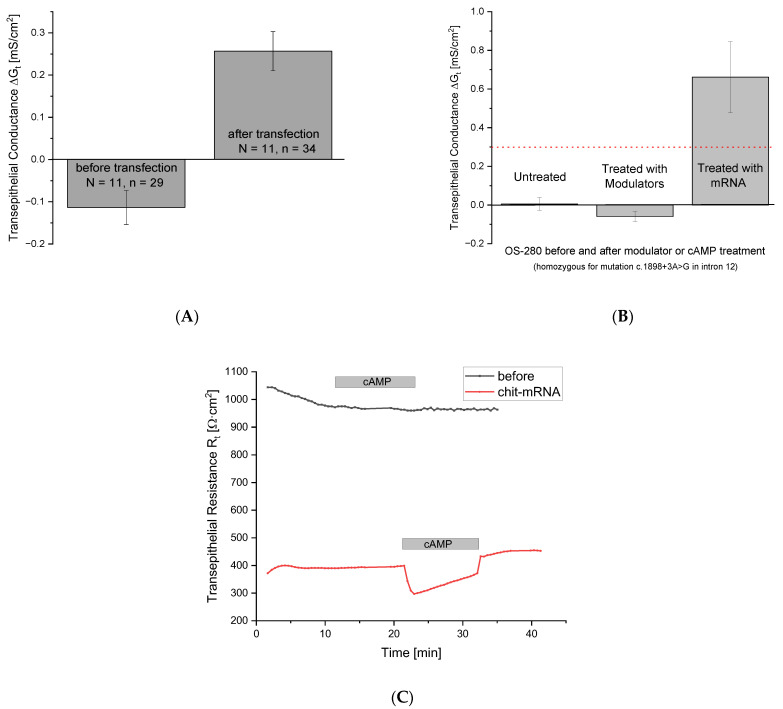
Functional CFTR restoration after wtCFTR-mRNA transfection. (**A**) Cells from pwCF were measured and subsequently incubated with wtCFTR-mRNA-chitosan-NCs. Before transfections all CF cells exhibited quite low G_t_ values (left bar) that turned to higher values as almost seen in HCs (right bar). (**B**) Comparison between modulator treatment and mRNA transfection. Cells from pwCF with Class 1 variants did not respond to modulator treatment, yet CFTR function was restored by wtCFTR-mRNA transfection using chitosan NCs. (**C**) Time course of a typical experiment before and after transfection with wtCFTR-mRNA-chitosan-NCs. Before transfection, CF cells show no answer to cAMP activation (black trace), whereas after transfection with wtCFTR-mRNA-chitosan NCs, the cells respond to cAMP activation with a marked decrease in R_t_, demonstrating restoration of the defective CFTR.

The incorporation of 5’ Cap 1 into in vitro-transcribed mRNA enhances transcript stability, translation efficiency and cellular tolerance by allowing efficient ribosome recruitment and reducing innate immune recognition at mRNA 5′-end [21]. Additionally, co-transcriptional 5′ Cap 1 capping decreases recognition by pattern-recognition receptors, hence increasing translation efficiency. Moreover, optimised 5′-end capping protects mRNA from degradation due to exonuclease activity eventually contributing to increased functional half-life and promoting robust transient protein expression. Chitosan-based delivery systems offer several advantages for mRNA transfection [10,22,23]. These include improved cellular uptake, protection of the transcript from extracellular degradation and reduced cytotoxicity compared to many synthetic polymers [11,23]. Due to its positive charge, the biopolymer chitosan forms stable nanoparticles that facilitate endocytosis when complexing mRNA, while its biodegradable and biocompatible properties support high transfection efficiency with minimal adverse cellular responses. We therefore assessed the effects of transfecting cells from pwCF with chitosan-wtCFTR-mRNA NCs (Figure 4A).

Prior to transfection, cells from pwCF had a cAMP-dependent ΔG_t_ of −0.01135 ± 0.04027 mS/cm^2^ (N = 11, n = 29), whereas after transfection with our wtCFTR-mRNA NCs, they had a ΔG_t_ of 0.2566 ± 0.04621 mS/cm^2^ (N = 11, n = 34). This increase in ΔG_t_ is only slightly below the level of HCs, and it is expected that further modifications of the wtCFTR-mRNA and the transfection protocol will result in much higher expression rates for the CFTR. However, in the case of a patient with a CFTR variant (homozygous for c.1898+3A>G in intron 12) that is not suitable for treatment with modulators, the expression rate is significantly higher; in a pilot experiment ΔG_t_ increased from close to zero to approx. 0.662 mS/cm^2^, while treatment with modulators showed no effect (Figure 4B).

The differences in R_t_ are very clear in a typical transfection experiment. Prior to transfection, CFTR-defective epithelial cells showed no response to cAMP stimulation, as indicated by stable R_t_. Following transfection with wtCFTR-mRNA-chitosan NCs, however, cAMP activation induced a pronounced decrease in R_t_, indicating the restoration of CFTR activity (Figure 4C).

**Figure 5:**
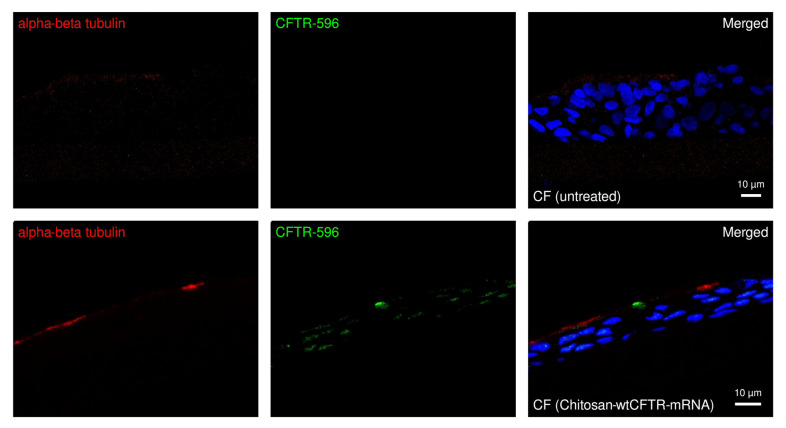
CFTR expression following chitosan-wtCFTR-mRNA transfection in ALI cultures. Representative immunofluorescence images of ALI cultures showing α/β-tubulin (red, 1:500) as an apical ciliary marker, CFTR-596 (green, 1:250), and nuclei (Hoechst, blue, 1:1000). Untreated cultures show minimal CFTR signal, whereas post-24 h transfection with chitosan-mediated wtCFTR-mRNA, a clear increase in CFTR signal is observed at the apical membrane. Scale bars are 10 µm.

To corroborate and further expand our electrophysiological findings, we used cryosections of the same ALI filters, after the Ussing chamber measurements for IF stainings with antibodies against alpha-beta tubulin (α/β-tubulin), as an apical marker and CFTR (Figure 5). Before mRNA transfection, no CFTR signal is visible in the cells (Figure 5, upper panel). However, after mRNA transfection, a clear apical signal for the CFTR is visible (Figure 5, lower panel). These results demonstrate that wtCFTR-mRNA transfection via chitosan delivery can restore proper localisation and function independently of the patient’s genetic CFTR profile.

### 2.5. Effects of CFTR Restoration on Claudin-1 Expression

**Figure 6:**
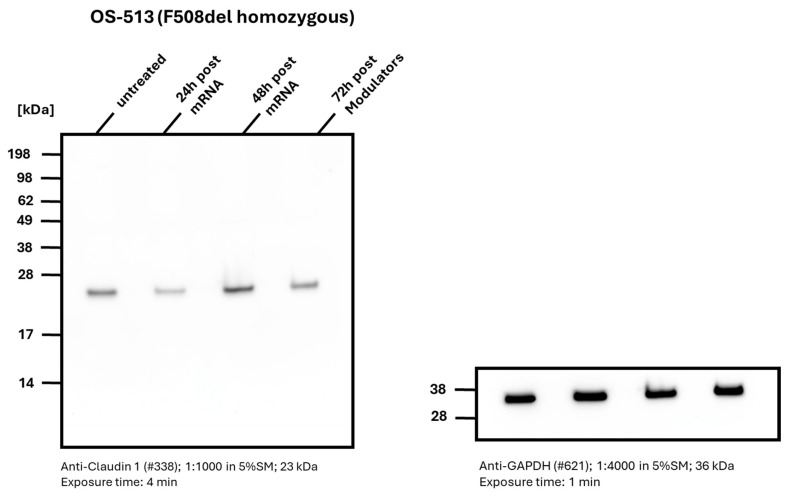
Western blot analysis demonstrates different expression levels of claudin-1 in a patient with biallelic F508del variants. In native condition, a stronger claudin-1 band is observed compared to that seen after 24 h transfection with chitosan-wtCFTR-mRNA, corroborating with lower R_t_, as observed in our electrophysiological measurements. The effect on claudin-1 expression disappeared after 48 h, which correlates with electrophysiological measurements showing an increase in R_t_. Modulator treatment resulted in claudin-1 levels comparable to cells from pwCF after 72 h. Equal protein loading was confirmed by comparable GAPDH band intensities. Quantitative densitometric data for the Western blot are included in Supplementary Materials, S2.

In a series of experiments involving the transfection of cells from pwCF (F508del homozygous) with chitosan-wtCFTR-mRNA NCs, we examined claudin-1 expression using Western blotting (Figure 6). Native cells from pwCF exhibited a strong claudin-1 band at 23 kDa, corresponding to higher R_t_ values measured by MTECC. Following 24 h after transfection of cells from pwCF with chitosan-wtCFTR-mRNA NCs, claudin-1 levels were reduced, akin to that observed in HC (see Figure 1C). After 48 h, claudin-1 band intensity and R_t_ values returned to levels like those of native cells treated with CFTR modulators for 72 h resulting in a moderate reduction in both claudin-1 expression and R_t_, although the effect was less pronounced than that observed 24 h post-wtCFTR-mRNA transfection. GAPDH signals were consistent across all samples, confirming equal protein loading. Densitometric analysis confirming Western blot results is included in the Supplementary Materials (Supplementary Materials, S2).

### 2.6. Viscosity Measurements

**Figure 7:**
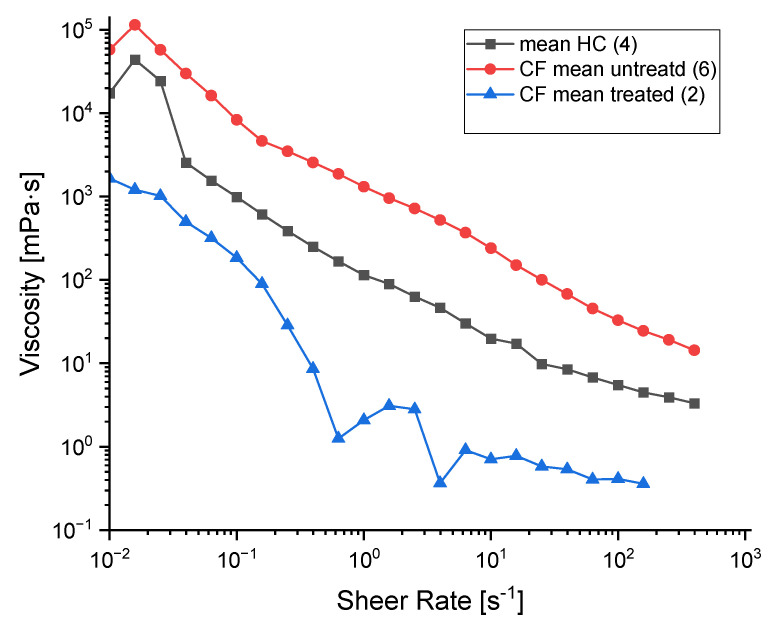
Relationship between shear rate and viscosity for cells from healthy control (HC), patients with cystic fibrosis (pwCF) and pwCF cells treated by modulators.

For comparison, typical viscosities of mucus of cells from HC, pwCF and from pwCF treated with elexacaftor and tezacaftor are given in Figure 7. The steady shear viscosity decreases through the entire range of explored shear rates for all samples, confirming their shear-thinning property. This characteristic is essential for mucus function as it enables the substance to stay as a protective gel-like layer under low stress and a more fluid state when exposed to higher shear forces, such as those that occur during coughing or clearance. To estimate the shear-thinning fluid properties of mucus quantitatively, experimental data measured in the rheometer were fitted using the power-law model, defined as:

(1) τ=K(γ.)^n

where τ is shear stress, $\dot{\gamma }$ is shear rate, K is the consistency index and n is the power-law index.

As observed in the curves, the viscosity values differ depending on the sample analysed. The power-law model provides consistency (K) and power-law (n) index, which represent viscosity and shear-thinning, respectively.

The viscosity of the mucus varies according to the studied system, as observed by the K values: the HCs showed the value of 2.3 Pa∙s, essentially lower than the untreated CF mucus samples, which exhibit a value of 3.2 Pa∙s. The observed behaviour is in accordance with the literature, as an increase in viscosity is described for pwCF [17]. Nevertheless, after treatment with modulators, a significant decrease in viscosity is accomplished (K value of 1.0 Pa∙s), which is evidence that the treatment was effective, with an efficient hydration of the mucus. In addition, for all the samples, the power-law index n [14] was lower than 1, which agrees with the observed shear-thinning characteristic.

## 3. Discussion

### 3.1. Multi Transepithelial Current Clamp (MTECC) System

The MTECC system is a technically advanced development of the conventional Ussing chamber that is specifically optimised for the functional analysis of epithelial monolayers derived from human airway cultures. Unlike traditional single-sample Ussing setups, the MTECC system can record simultaneously from up to four epithelial layers, enabling the parallel evaluation of multiple experimental conditions under identical environmental parameters. The system integrates the continuous, real-time measurement of the PD, R_t_ and equivalent short-circuit current (I_eq_) within a temperature- and gas-controlled environment. This maintains physiological conditions throughout the experiment.

MTECC also incorporates low-frequency sinusoidal current application and Fourier-based impedance analysis. This markedly improves signal stability and reduces electrical noise compared to classical stepwise voltage clamp protocols. Its compact, semi-sterile design enables the same ALI cultures to be used repeatedly before and after pharmacological interventions, facilitating longitudinal CFTR-functional testing in patient-derived epithelia. The system’s high reproducibility, sensitivity and throughput enable the detection of subtle CFTR-dependent conductance changes that may be missed in conventional setups [14].

Overall, the MTECC platform provides a more efficient, precise and physiologically relevant method of evaluating ion transport and modulator responsiveness in differentiated human epithelial cultures, making it ideal for personalised CF diagnostics and therapy-typing studies.

### 3.2. Variant-Dependent Responses to CFTR Modulator Therapy

Our results demonstrate a clear variant-dependent response to CFTR modulators in patient-derived nasal epithelial ALI cultures. In samples harbouring modulator-responsive variants (F508del homozygous) treatment with elexacaftor and tezacaftor for 72 h followed by acute exposure to ivacaftor resulted in robust CFTR restoration in CFTR-mediated ion transport, based on electrophysiological measurements. This functional recovery was further supported by increased apical CFTR signal assessed with IF and a reduction in MUC5AC expression following modulator treatment indicating not only channel rescue but also downstream improvement of epithelial homeostasis. Restoration of CFTR function was accompanied by a reduction in MUC5AC, a gel-forming mucin that contributes substantially to mucus viscoelasticity. In CF airway epithelia, elevated MUC5AC expression, together with impaired ion and water transport, promotes mucus dehydration and increased viscosity. Improved CFTR-mediated ion transport is therefore expected to enhance airway surface hydration and reduce goblet-cell-associated MUC5AC accumulation, providing a mechanistic link between reduced MUC5AC levels and the observed improvement in mucus hydration.

In contrast, patients with non-responsive variants show minimal or absent functional restoration upon modulator treatment. This underscores the genotype-dependent mechanism of current CFTR modulator therapies. Importantly, the observed reduction in MUC5AC levels following modulator treatment in responsive variants suggests that restored CFTR activity may indirectly modulate mucus production or secretion, potentially through improved airway surface hydration and altered epithelial differentiation. Although CFTR modulators are not designed to directly target mucin expression, our findings support the concept that functional rescue of ion transport can translate into measurable changes in mucus-associated phenotypes, which are clinically relevant hallmarks of CF.

### 3.3. Association Between CFTR Functional Rescue Post-Modulator Treatment and Mucus-Associated Phenotypes

Altered mucus rheology is a key consequence of impaired CFTR function and represents a major determinant of mucociliary clearance failure in CF. Several rheological studies have demonstrated that CF airway mucus exhibits markedly increased viscosity across a wide range of shear rates, particularly under low-shear conditions relevant to normal breathing, compared to HC [17]. Importantly, these studies also show that therapeutic interventions capable of improving epithelial hydration are associated with a clear reduction in mucus viscosity, partially restoring shear-thinning behaviour toward a non-CF phenotype.

In line with these observations, ALI cultures from pwCF that were treated with elexacaftor and tezacaftor, specifically those with modulator-responsive genetic variants, exhibited not only functional restoration of CFTR-mediated ion transport, but also molecular changes associated with mucus normalisation, including reduced MUC5AC expression. Although mucus rheology was assessed independently, the observed reduction in mucin levels following modulator treatment links restored chloride transport, improved airway surface hydration, and decreased mucus viscosity. This is consistent with rheological profiles reported for CF samples that were treated with modulators, which demonstrates a shift toward lower viscosity values, particularly at physiologically relevant shear rates.

By contrast, cultures harbouring non-responsive genetic CFTR variants showed minimal electrophysiological improvement following modulator treatment along with no reductions in mucus-associated markers like the MUC5AC protein. This suggests that, in modulator non-responsive variants, abnormal mucus rheology is likely to persist despite modulator exposure, reinforcing the concept that effective CFTR restoration is a prerequisite for complementary improvements in mucus biophysical properties.

Together, these findings support a functional cascade model in which CFTR modulators restore epithelial ion transport, leading to downstream effects on mucus composition and biophysical behaviour. While CFTR modulators do not directly target mucins or mucus structure, our data interpretation establishes rheological evidence indicating that successful CFTR correction can indirectly normalise mucus properties, which is of high clinical relevance.

Future studies integrating direct rheological measurements with electrophysiological and molecular readouts in the same patient-derived cultures would provide a powerful framework for linking patient-specific CFTR function to mucus rheology. Such an approach may be particularly valuable for evaluating emerging therapies, where improvements in mucus viscosity may serve as an early functional indicator of therapeutic efficacy.

### 3.4. mRNA-Based wtCFTR Delivery as a Genotype-Independent Therapeutic Strategy

Beyond CFTR modulators, mRNA-based therapeutic approaches offer variant-independent strategies for restoring CFTR function, especially for patients with modulator-non-responsive variants [24]. In our study, wtCFTR-mRNA synthesised by in vitro transcription and co-transcriptionally capped was used to transiently express the CFTR in CF patient-derived airway epithelial cultures [25]. Cap-1 incorporation is critical for efficient translation and mRNA stability and is known to reduce innate immune response at the 5′ end, thereby improving protein expression in epithelial cells. Our findings indicate that mRNA-mediated CFTR expression bypasses the underlying genetic defect which is particularly relevant in pwCF harbouring non-responsive CFTR variants where modulator combinations fail to restore sufficient CFTR channel activity. This highlights the potential of mRNA therapy as an alternative therapeutic strategy for pwCF. Moreover, enhanced translational efficiency due to optimised 5′ capping is especially advantageous in airway epithelia, where CFTR-expressing cell populations are limited and relatively high local protein expression is crucial to attain measurable functional rescue [26]. Within this context, wtCFTR-mRNA delivery enables rapid, controllable CFTR expression without permanent genomic modification, making it suitable for repeatable functional testing in patient-derived airway models. This aligns well with our experimental platform, which allows repeated electrophysiological measurements on the same ALI cultures, and facilitates direct comparison between modulators and mRNA-based therapeutic strategies.

Taken together, current CFTR modulator therapies are variant-specific and depend on the presence of an endogenous CFTR protein, restricting their application to certain genetic variants. However, mRNA-based therapeutic approaches offer a genotype-independent strategy, delivering a full-length wtCFTR transcript that enables functional CFTR expression regardless of the underlying genotype. This approach could overcome limitations associated with defective CFTR biosynthesis, nonsense-mediated mRNA decay and severe protein misfolding. It could also facilitate a more comprehensive restoration of CFTR-mediated anion transport, including both chloride and bicarbonate. Unlike modulator therapies, which generally provide partial functional correction and require continuous administration, mRNA therapy enables transient and controllable CFTR expression without permanent genomic modification. Despite challenges related to delivery efficiency, durability and immune responses remain; wtCFTR-mRNA therapy has the potential to address several biological limitations of current modulator-based treatments and warrants further investigation as an alternative therapeutic strategy for CF.

### 3.5. Limitations of mRNA-Based Approaches

Despite the promising functional rescue observed in vitro, there are several challenges that must be addressed before nebulised chitosan-mRNA delivery can be translated into clinical practice [27]. These include ensuring aerosol stability and homogeneous airway deposition, overcoming the challenge of thick CF mucus and mucociliary clearance, managing the transient nature of mRNA expression which requires repeated dosing, and carefully evaluating the long-term safety of repeated airway exposure. Therefore, optimising the carrier formulation, dosing strategies, and conducting in vivo validation will be essential for successful clinical implementation. Because mRNA expression is transient by nature, repeated administration to maintain therapeutic levels of CFTR expression is required. The attenuation of mRNA-mediated effects observed between 24 and 48 h is consistent with the transient expression nature of exogenously delivered mRNA, which is inherently limited by mRNA stability, protein turnover, and epithelial homeostatic regulation. While early time points demonstrated robust CFTR functional rescue and associated epithelial responses, sustained effects are likely to require optimised dosing strategies. In addition, efficient mRNA delivery across differentiated airway epithelia remains challenging, especially in the presence of thick mucus and altered epithelial barriers in CF. Such barriers can substantially limit nanoparticle penetration and cellular uptake of nucleic acid-based therapeutics.

In this context, mucoadhesive delivery systems such as chitosan-based carriers may provide partial advantages, as chitosan has been shown to interact with mucins and extend residence time at the epithelial surface. These properties may be particularly relevant in CF airways, where excessive mucus accumulation together with impaired clearance restrict access of therapeutic agents to the epithelial surface [10,28]. However, excessive mucoadhesion may also hinder deeper penetration through dense mucus layers, emphasising the need for careful optimisation of carrier composition and physicochemical properties.

Furthermore, although capping of wtCFTR-mRNA reduces innate immune activation, the long-term effects of repeated airway exposure remain insufficiently characterised and require careful evaluation. In the present study, wtCFTR-mRNA delivery was performed under controlled in vitro conditions, which may not fully capture the complexities of in vivo airway delivery, including mucociliary clearance, immune surveillance, and mechanical forces. Therefore, further optimisation of delivery vehicles, including dosing strategies and administration schedules, is crucial for direct clinical translation of these findings.

In differentiated airway epithelial cells grown under ALI conditions, the durability of modified wtCFTR-mRNA expression is likely influenced by intracellular mRNA stability, translational persistence, and the turnover rate of newly synthesised CFTR protein rather than by delivery efficiency [29]. Further improvements may be achieved by optimising untranslated regions and codon usage to reduce intracellular mRNA decay. In addition, prolonging the functional half-life of apically expressed CFTR protein may contribute to sustained electrophysiological rescue, even after mRNA levels decline. From a translational perspective, modest extensions in intracellular mRNA or protein stability could meaningfully reduce dosing frequency in vivo, which is particularly relevant for chronic airway delivery and clinical feasibility of repeated administration.

Compared with approaches using LNPs to deliver wtCFTR-mRNA to the lungs, which are limited by mucus penetration barriers, variable epithelial targeting, and the potential to activate the innate immune system, our chitosan-mediated delivery achieved robust functional rescue in primary-acute-lung-injury ALI cultures, including those with modulator-non-responsive variants (Figure 4). Importantly, CFTR restoration was accompanied by improvements in epithelial barrier properties and mucus-associated phenotypes, indicating structural and functional recovery beyond ion transport. However, as with other mRNA-based strategies, expression remains transient and requires optimisation for long-term clinical use.

Although chitosan-mediated mRNA delivery resulted in marked functional and morphological improvement, CFTR rescue did not fully reach levels observed in HC cultures. Future optimisation strategies may include refinement of mRNA design, such as incorporation of modified nucleosides (e.g., pseudouridine) to enhance stability and translational efficiency, as well as optimisation of dosing frequency, with the aim of improving the magnitude and durability of CFTR rescue in patient-derived airway epithelial models.

### 3.6. Future Perspectives Towards Personalised CF Therapy

Looking ahead, mRNA-based CFTR delivery could be integrated into personalised therapeutic pipelines, particularly for individuals who show limited benefits from existing CFTR modulators. Combining ex vivo functional screening with patient-derived ALI cultures may allow classification of patients according to their responsiveness to modulators versus wtCFTR-mRNA-based restoration. Moreover, complementary assessment of downstream phenotypes, such as mucus composition and rheology, could provide additional functional endpoints to evaluate therapeutic efficacy.

Ultimately, the convergence of CFTR modulators, mRNA therapeutics, and advanced epithelial model systems may show additional effects when therapies are combined or enable a more comprehensive and personalised treatment strategy for CF, addressing both the molecular defect and its physiological consequences.

### 3.7. CF Tissues Exhibit Altered Tight Junction Organisation

Our findings demonstrate that CF-derived airway epithelial ALI cultures exhibit significantly higher R_t_ than HCs, accompanied by reduced G_t_ and increased claudin-1 expression. These results suggest that the elevated R_t_ observed in CF epithelia reflects altered paracellular ion permeability rather than an improvement in the quality of the epithelial barrier. Claudin-1 acts as a sealing claudin that restricts paracellular ion flux. Its upregulation has previously been associated with increased epithelial tightness and higher R_t_ [30].

In the context of CF, increased claudin-1 expression may represent an adaptive or compensatory response to impaired transcellular ion transport caused by defective CFTR function. This results in a redistribution of epithelial ion handling toward tighter junctional regulation. Importantly, transiently reducing claudin-1 expression following CFTR restoration by mRNA transfection, together with normalisation of electrophysiological parameters, supports the concept that CFTR activity is functionally linked to tight junction organisation and paracellular ion selectivity [13]. These observations underscore the fact that epithelial barrier alterations in CF are dynamic and closely coupled to CFTR-dependent ion transport, rather than being fixed structural abnormalities.

Strikingly, transfection of cells from pwCF with chitosan-wtCFTR-mRNA NCs led to transient normalisation of electrophysiological parameters followed by CFTR restoration and reduction in claudin-1 expression. This further supports a functional link between CFTR activity, tight junction organisation and paracellular ion selectivity [13]. These findings together suggest that tight junction alterations in CF airway epithelia are dynamic and reversible and are closely associated with CFTR-dependent ion transport and epithelial homeostasis.

## 4. Materials and Methods

### 4.1. Patient Recruitment and Ethical Considerations

The study was performed at the University Children’s Hospital, Münster, with ethical approval provided by the local institutional review board (Ethik-Kommission der Ärztekammer Westfalen-Lippe und Universität Münster, No. 2014-585-f-S). Nasal epithelial samples were collected from participants during routine outpatient appointments, following written informed consent from all subjects or their guardians. Inclusion criteria required a confirmed clinical diagnosis of CF based on biallelic CFTR pathogenic variants classified according to ACMG consensus guidelines. All procedures conformed to the principles outlined in the Declaration of Helsinki. Patient-derived samples were assigned to CFTR-modulator- or mRNA-based rescue experiments based on random cohort allocation determined by the availability of newly established ALI cultures, rather than by pre-selection of specific genotypes. This strategy minimised selection bias while ensuring efficient use of limited patient-derived primary cultures. Samples were excluded if no genetically confirmed CFTR variant status was available, the clinical presentation or diagnostic documentation was incomplete or inconsistent, or the epithelial differentiation under ALI conditions was insufficient (e.g., poor ciliation or low baseline transepithelial resistance (below 100 Ω∙cm^2^)).

### 4.2. ALI Cell Culture

Human nasal cells were obtained and cultured as previously described [14]. Briefly, primary nasal epithelial cells acquired via brushing were cultured in RPMI medium with 2% antibiotic/antimycotic at 37 °C and 5% CO_2_ in humidified conditions. Following initial outgrowth in collagen-coated T25 flasks, cells were seeded onto collagen-coated Costar Transwell^®^ inserts (diameter 6.5 mm, Corning, NY, USA) at 100,000 cells per filter, using PneumaCult™-Ex Medium (STEMCELL Technologies, Vancouver, BC, Canada) for expansion. Once confluent, cells were transitioned to ALI conditions and fed with PneumaCult™-ALI Maintenance Medium on the basolateral side every 2–3 days. Differentiation and ciliation were allowed for at least 21–30 days before downstream analyses. Both HC and CF samples underwent identical protocols.

### 4.3. Cryosection Preparation for IF Stainings

Transwell-grown ALI cultures were excised and embedded in frozen section medium (Tissue-Tek Cryomold (Tokyo, Japan) and Shandon Cryomatrix (Melville, NY, USA)). The resulting blocks were sectioned at 20 µm using a Leica CM3050S cryostat (Leica, Nussloch, Germany) and transferred onto charged microscope slides (SuperFrost Plus, Thermo Scientific, Waltham, MA, USA) for further staining.

### 4.4. IF Stainings

Cell sections were rinsed in phosphate-buffered saline (PBS), fixed for 15 min in 4% paraformaldehyde, permeabilised in 0.2% Triton X-100, and blocked for 2 h in 2% bovine serum albumin (BSA) and 5% goat serum PBS. For CFTR IF stainings, sections of ALI filters were incubated overnight at 4 °C with primary antibodies—anti-αβ-tubulin (1:1000, Cell Signalling Technology, Danvers, MA, USA) and anti-CFTR (1:250, UNC Antibody Distribution Program, Chapel Hill, NC, USA)—diluted in blocking buffer. After washing, Alexa Fluor-conjugated secondary antibodies (488 anti-mouse, 546 anti-rabbit, Invitrogen; both 1:1000) were applied for 1 h at room temperature (RT), followed by Hoechst 33342 nuclear staining (1:1000 in PBS, Sigma, St. Louis, MO, USA). Following the same protocol, MUC5AC immunostaining was performed with primary antibody-anti-MUC5AC (1:500, Thermo-Fischer MA5-12178, Waltham, MA, USA). After washing, Alexa Fluor-conjugated secondary antibodies (488 anti-mouse, 546 anti-rabbit, Invitrogen, Carlsbad, CA, USA; both 1:1000) were applied. Slides were then mounted in DAKO fluorescent medium and imaged with Zeiss LSM 880 confocal microscopy (Jena, Germany). Z-stack images were processed with ZEN software (V3.2). MUC5AC fluorescence intensity was quantified using Fiji (ImageJ v2.16.0). Z-stack images were converted to maximum-intensity projections, and regions of interest were manually defined over MUC5AC-positive areas (green signal) along the apical epithelial layer. Mean fluorescence intensity was measured after background subtraction, and values from multiple regions per sample were averaged. Imaging was performed using single-channel acquisition with identical exposure settings. Image post-processing in OMERO was limited to uniform brightness/contrast adjustments applied equally to entire images without altering relative signal patterns.

### 4.5. Transepithelial Ion Transport Measurements (MTECC Ussing Chamber)

Electrophysiological assessment of epithelial function was conducted on ALI cultured cells using the MTECC system, as previously described [14]. Each experiment involved mounting up to four Transwell inserts in a multichannel chamber filled with Ringer’s solution at 37 °C. Open circuit potential and R_t_ were recorded via a four-electrode arrangement. After baseline stabilisation, cAMP-IBMX (8-[4-chlorophenylthio (CTP)]-cAMP (100 μM; Biolog, Hayward, CA, USA), IBMX (1 mM; Sigma-Aldrich, St. Louis, MO, USA)) cocktail was added basolateral to stimulate CFTR activity, and subsequent changes in current were digitised and analysed. Data were normalised to the membrane area and presented as mean ± SEM.

### 4.6. CFTR Modulator Incubation

Pre-incubation of pwCF ALI cultures with elexacaftor and tezacaftor (each 3 μM, 72 h) preceded functional analysis. During the MTECC measurements, ivacaftor (1 μM) was acutely added to both apical and basolateral compartments. Reagent sources included MedChemExpress unless otherwise specified. Modulator concentrations adhered to previous reports of safety and efficacy [31].

### 4.7. Preparation of wtCFTR-mRNA and Chitosan Nanoparticles

Chitosan was purchased from HMC+ GmbH (Halle/Saale, Germany) as an ultrapure biomedical-grade sample (Heppe 70/5; Batch-No. 212-170614-01; DA = 17%; Mw = 29.3%). It was dissolved in water with 5% stoichiometric excess of 5 M HCl. The NCs were prepared as described previously [22]. Chitosan-mRNA complexes were prepared at an N/P ratio of 75. Complexes were formulated in Opti-MEM and applied apically to ALI cultures. No nebulization/aerosolization was performed; delivery was achieved by direct apical administration to the ALI filters. Briefly, 400 µL ethanolic lecithin solution (100 mg/mL) was mixed with 530 µL of ethanol and further supplemented with 125 µL Miglyol^®^ 812 N and 9.5 mL ethanol. Under constant stirring, this solution was poured into 20 mL aqueous Chitosan (CS) solution (0.5 mg/mL). Concentration of the milky mixture was performed at 40 °C, using a rotavapor (Büchi R-210, Büchi Labortechnik GmbH, Essen, Germany) and in this process, the pressure was slowly decreased to 0 mbar, until 3.5–4.0 mL remained. Finally, the volume was topped up to 4 mL with water to yield a final CS concentration of 2.5 mg/mL.

To prepare in vitro-transcribed mRNA (IVT mRNA), the plasmid was linearised using the SalI restriction enzyme and used as a template for in vitro transcription. IVT mRNA corresponding to the CFTR open reading frame was synthesised using the HiScribe^®^ T7 High Yield RNA Synthesis Kit (E2040S; New England, Biolabs, Ipswich, MA, USA), with co-transcriptional incorporation of a Cap 1 structure using TriLink CleanCap^®^ AG. Following transcription, IVT mRNA was purified using a RNeasy Mini Kit (Qiagen, Venlo, The Netherlands) according to the manufacturer’s instructions. The concentration and purity of the synthesised mRNA were assessed using a NanoDrop™ One spectrophotometer (Thermo Fisher Scientific, Waltham, MA, USA).

The chitosan NCs were loaded with wtCFTR-mRNA by carefully mixing 1 µL of chitosan NCs with wtCFTR-mRNA (0.3 µg/ALI filter) followed by adding nuclease-free water up to 50 µL at RT for 30 min, to allow complexation of the mRNA and the chitosan. Further on, 50 µL of Opti-MEM™ Reduced Serum Medium (Gibco, Thermo Fisher Scientific, Waltham, MA, USA) was added to the complex solution and incubated for a further 5 min at RT. In the meantime, ALI cultures were gently washed with PBS from the apical side. Finally, chitosan-wtCFTR-mRNA NCs (100 µL) are added to the apical side of the ALI filters and incubated for 24 h at 37 °C, 5% CO_2_ and 95% rH.

### 4.8. Viscosity Measurements

Mucus collections from the apical side of ALI cultures were gently incubated for a minute with 50 µL of PBS in each ALI filter followed by collection in Eppendorf tubes and analysed using a rheometer to measure viscosity. The viscosity of the mucus was determined with a rheometer (Anton Paar MCR 302, Graz, Austria). The cone plate geometry is 25 mm in diameter and has an angle of 1°, resulting in a gap of 49 µm at the cone edge. This geometry advantageously requires a small sample volume (70 µL). Flow properties were assessed as a function of shear stress from 0.01 to 1000 s^−1^ at 37 °C.

### 4.9. Western Blotting

Proteins were mixed with 4× Lithium Dodecyl Sulphate (LDS) sample buffer and 1M Dithiothreitol (DTT) and heated at 70 °C for 10 min prior to loading onto a NuPAGE 10% Bis-Tris gel with MES buffer. After electrophoresis, proteins were transferred onto a PVDF membrane at 30 V for 3 h on ice. The membrane was blocked for 1 h at room temperature with 5% skim milk (SM) prepared in PBS-T (PBS with 0.1% Tween-20), followed by overnight immunoblotting at 4 °C with rabbit polyclonal antibody against claudin 1 (Abcam, ab15098; 1:1000) or rabbit monoclonal antibody against GAPDH (Cell Signaling Technology, #2118; 1:4000, Danvers, MA, USA), diluted in the same blocking solution. Membranes were washed three times with PBS-T for 10 min each, followed by incubation for 1 h at RT with a horseradish peroxidase (HRP)-conjugated donkey anti-rabbit secondary antibody (Cytiva Cat# NA934, RRID: AB 772206; 1:3000) diluted in 5% SM and PBS-T. Following a second series of PBS-T washes, protein bands were visualised using the ECL Prime Western Blotting Detection Reagent (GE Healthcare Life Sciences, Chalfont St Giles, UK). Chemiluminescent signals were recorded with the FUSION-SL 3500WL Advance Imager (PeqLab, VWR International, Radnor, PA, USA) and processed using Adobe Illustrator CS4, V. 9.0.

### 4.10. Statistical Analysis

Statistical comparisons and data visualisation were performed in OriginPro 2025^®^ (OriginLab). Both biological replicates (individual patient donors, N) and technical replicates (filter inserts, n) were specified. Data are reported as mean values ± SEM, and statistical significance was defined as *p* < 0.05 unless otherwise indicated. Paired comparisons (e.g., before and after treatment within the same donor-derived ALI cultures) were performed using paired Student’s *t*-tests in OriginPro 2025^®^. We defined statistical significance as *p* < 0.05; however, most of the reported comparisons reached higher levels of significance (e.g., *p* ≤ 0.01 or *p* ≤ 0.001).

## 5. Conclusions

This work establishes a unified functional–structural evaluation framework that integrates a modified Ussing chamber system MTECC with molecular and morphological analyses in patient-derived airway epithelial ALI cultures. The MTECC permits repeatable, longitudinal measurement of CFTR activity in intact epithelial preparations, enabling reliable discrimination of variant-dependent responses to CFTR modulators as well as identification of functionally non-responsive phenotypes unlikely to benefit from existing modulator therapies. Combining electrophysiological outputs with CFTR protein localisation and mucus-associated markers allows functional defects to be interpreted within their cellular and biophysical context, supporting the application of ex vivo theratyping, a clinically applicable approach for individualised treatment decisions in cystic fibrosis. Critically, this strategy captures inter-patient functional heterogeneity that is not resolved by genotype alone, including among patients carrying similar or modulator non-responsive variants. In parallel, CFTR delivery via co-transcriptionally capped (Cap-1) wtCFTR-mRNA demonstrates promising mutation-independent therapeutic modalities as an alternative to cases non-responsive to modulator therapy. Within this experimental framework, patient-specific CFTR functional and structural profiling provides a versatile preclinical platform for systematic evaluation of emerging therapies, including mRNA-based interventions. Collectively, these results position integrated electrophysiology, functional theratyping, and advanced molecular therapeutic strategies as complementary components of a function-driven, patient-tailored treatment strategy for cystic fibrosis.

ALI cultures from pwCF, along with measurements of CFTR activity using the MTECC system, provide a comprehensive experimental framework for theratyping in a physiologically relevant context. These multi-method approaches allow integration of patient-specific epithelial phenotypes into the field of personalised medicine, complementing genotype-based classification and direct CFTR functional evaluation. Overall, within this translational pipeline, CFTR-mRNA based therapies represent a potential genotype-independent strategy that can be rigorously assessed and refined using MTECC-based theratyping platforms.

## Data Availability

The original contributions presented in this study are included in the article/Supplementary Material. Further inquiries can be directed to the corresponding author.

## Supplementary Materials

The following supporting information can be downloaded at: https://www.mdpi.com/article/10.3390/ijms27042063/s1.
