# Supplementary material for: Restoration of Defective CFTR in Human Nasal Respiratory Epithelial Cells by CFTR Modulators and mRNA Transfection
Source: Int J Mol Sci. 2026 Feb 23;27(4):2063. doi: 10.3390/ijms27042063 (PMC12940240; doi:10.3390/ijms27042063)
Supplement: Supplementary file 1 [file ijms-27-02063-s001.zip › Supplement S1.pdf]

Supplement S1

Table S1. Genetic variants of CF cases (HGVS protein-level nomenclature)

Case No.	CFTR genotype (allele 1 / allele 2) – protein level (HGVS)	Sex	Age
1	p.Phe508del / p.Phe508del	M	51
2	p.Phe508del / p.Phe508del	M	26
3	p.Phe508del / p.Phe508del	M	25
4	p.Phe508del / p.Phe508del	F	10
5	p.Phe508del / p.Phe508del	F	19
6	p.Phe508del / p.Arg117His	F	36
7	p.Phe508del / p.Arg553*	F	37
8	p.Phe508del / p.Phe336Ile	M	24
9	p.Phe508del / p.Ile336Lys	M	37
10	p.Phe508del / p.Arg553*	F	36
11	p.Phe508del / p.Ile336Lys	F	1
12	p.Gly576Ala / p.Arg668Cys	F	76
13	p.Val201Met / p.(=)	F	46

14	p.Phe508del / p.Arg117His	F	1
15	p.Phe336Ile / p.Phe336Ile	M	32
16	p.Phe508del / p.(=)	M	37
17	p.Leu715Phefs*5 / p.Glu1047_Val1122del	F	6
18	p.Ser1176_Gly1179del / p.Ser1176_Gly1179del	F	11
19	p.Phe508del / p.Lys728Serfs*6	M	34
20	p.Val201Met / p.Val201Met	F	34
21	p.Phe508del / p.Phe508	F	3
