# Supplementary material for: Restoration of Defective CFTR in Human Nasal Respiratory Epithelial Cells by CFTR Modulators and mRNA Transfection
Source: Int J Mol Sci. 2026 Feb 23;27(4):2063. doi: 10.3390/ijms27042063 (PMC12940240; doi:10.3390/ijms27042063)
Supplement: Supplementary file 1 [file ijms-27-02063-s001.zip › Supplement S2.pdf]

Supplement S2

Densitometric Densities of Western Blots from Figure 1C and Figure 6

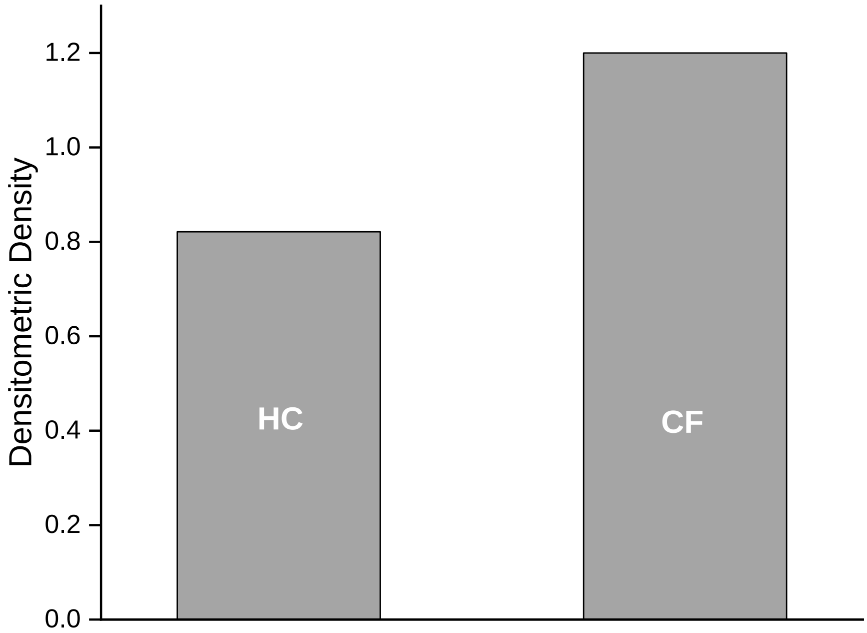

Supplement to Fig. 1C: Densitometric quantification of a Western Blot showing expression of claudin-1. Cells from a pwCF showed higher claudin-1 expression as compared to HC. Values are normalised to GAPDH expression (relative units).

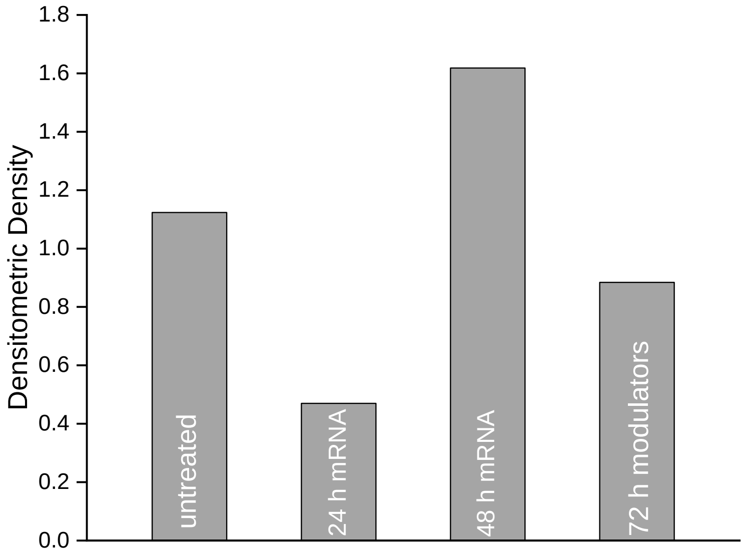

Supplement to Fig. 6: Densitometric quantification of a Western Blot showing expression of claudin-1 before and after wtCFTR-mRNA transfection and modulator incubation. Twenty-four hours after wtCFTR-mRNA transfection, cells derived from a responsive patient (F508del homozygous) exhibited lower claudin-1 expression than before transfection. This effect disappeared after 48 hours. Modulator treatment of cells from the same patient resulted in reduced claudin-1 expression, yet the effect was lower than that achieved by mRNA transfection. Values are normalised to GAPDH expression (relative units).
